# Association between tooth retention and lower inflammatory dietary pattern in older adults

**DOI:** 10.3389/fragi.2026.1945744

**Published:** 2026-10-02

**Authors:** Lyzbeth B. Ortiz-Barrios, Paola Garcia-delaTorre, Berenice Rivera-Paredez, Sergio Sánchez-García, Berenice Palacios-González

**Affiliations:** 1 Unidad de Investigación Epidemiológica y en Servicios de Salud, Área de Envejecimiento, Centro Médico Nacional SXXI, Instituto Mexicano del Seguro Social, Mexico; 2 Posgrado en Ciencias Médicas Odontológicas y de la Salud, Universidad Nacional Autónoma de Mexico, Mexico; 3 Centro de Investigación en Políticas, Población y Salud, Facultad de Medicina, Universidad Nacional Autónoma de México (UNAM), Mexico; 4 Laboratorio de Genómica del Envejecimiento del Instituto Nacional de Medicina Genómica (INMEGEN) en el Centro de Investigación sobre Envejecimiento (CIE-CINVESTAV Sede Sur), Mexico

**Keywords:** aging, dietary pattern, inflammatory potential, oral health, tooth loss

## Abstract

**Introduction:**

Tooth loss is highly prevalent among older adults and may compromise food selection, diet quality, and adherence to proinflammatory dietary patterns, thereby contributing to chronic inflammation and adverse health outcomes. This study investigated the association between tooth retention and the inflammatory potential of the diet among older adults.

**Methods:**

A cross-sectional analysis was conducted among 1,075 adults aged 60 years or older. According to the oral examination, participants were categorized as having ≥21, 11-20, 1-10, or no natural teeth, and as edentulous. The Dietary Inflammatory Index was constructed using a food-frequency questionnaire.

**Results:**

Individuals with more teeth were younger, possessed higher educational attainment, were less likely to smoke, and exhibited elevated Mini-Mental State Examination scores. After adjustment for potential confounders, older adults with 1–10 natural teeth had significantly higher odds of belonging to a more pro-inflammatory dietary category than those with 21–28 teeth (OR = 2.19; 95% CI: 1.44-3.33; p-value <0.001).

**Conclusion:**

Our findings suggest that tooth retention is closely associated with the inflammatory potential of the diet in older adults and supports the integration of oral health into nutritional assessment and healthy aging strategies.

## 1 Introduction

According to the World Dental Federation’s definition, oral health encompasses many capabilities, such as smiling, speaking, tasting, chewing, swallowing, and expressing emotions through facial gestures, all done safely and without pain (Huang et al., 2025). Tooth loss is an indicator of oral health status and reflects the history of oral conditions over a lifetime (Huang et al., 2025; Organization, 2022). The incidence of tooth loss rises, particularly among older adults (He et al., 2025). According to data from the World Health Organization, the global prevalence of tooth loss among older adults is estimated to be approximately 20% (Organization, 2022). A population-based study revealed that people with periodontal disease, cavities, or greater tooth loss had a higher risk of cardiovascular events (Park et al., 2019). Furthermore, a study revealed that for every additional tooth loss, the risk of cognitive decline (1.4%) and dementia (1.1%) increases (Qi et al., 2021). Oral health problems in older adults have been associated with malnutrition and sarcopenia (Ramos et al., 2022). Tooth loss has been associated with substantial dietary restrictions and reduced variety and nutrient intake in older adults (Anastassiadou and Heath, 2002; Moynihan and Varghese, 2022; Savoca et al., 2010). This can create a vicious cycle: poor oral health is associated with poor nutritional status, which, in turn, exacerbates oral problems and impairs general health. For example, Shen et al. reported lower dietary diversity and poorer nutritional status in older adults with poor dentition (Shen et al., 2023).

Nutrition research has focused on the effects of individual nutrients; however, people consume food and food combinations. Consequently, studying a single component fails to reflect the complexity of a dietary pattern and may lead to conclusions that do not necessarily reflect the diet. Adherence to a dietary pattern can be assessed using *a priori* and *a posteriori* methods (Khan et al., 2018). An *A priori* dietary pattern relies on predefined criteria: variables are quantified and summed to generate a score or index that measures diet quality. One such pattern is the DII. This index was developed to classify dietary patterns along a continuous scale ranging from anti-inflammatory to pro-inflammatory; it comprises energy macronutrients, micronutrients (vitamins and minerals), and polyphenols, with weightings assigned based on their pro-inflammatory or anti-inflammatory effects, as supported by evidence (Shivappa et al., 2014). Evidence indicates that higher DII scores are associated with periodontitis and a lower number of teeth (Moynihan and Varghese, 2022; Feng et al., 2022; Shi et al., 2025), suggesting that higher DII scores could be associated with an increased risk of tooth loss. Therefore, the aim of this study is to determine whether the number of remaining teeth and the DII index are associated in older Mexican adults.

## 2 Materials and methods

### 2.1 Study design and samples

This is a cross-sectional study using data from the 2019 Cohort of Obesity, Sarcopenia, and Frailty of Older Mexican Adults (COSFAMM, in Spanish). Briefly, COSFAMM is a population-based, prospective study that began in 2014 (with refreshments in 2019 and 2024), in which participants were randomly selected from the administrative records of a Mexican public healthcare institute. Clinical and sociodemographic information is collected annually through interviews conducted by trained staff. Each participant signed a consent form explaining the study procedures and benefits. From the initial sample of 1,677 older adults, 1,075 participants were included in the final analysis after excluding individuals who did not complete the interview or clinical oral evaluation, or who had an implausible/aberrant energy intake (<500 or > 6,500 kcal/day) or an incomplete food frequency questionnaire (FFQ). Hence, this study used data from 1,075 subjects, as shown in Figure 1. Supplementary Table S1 shows the characteristics of all participants (N = 1,677) without exclusions.

**FIGURE 1:**
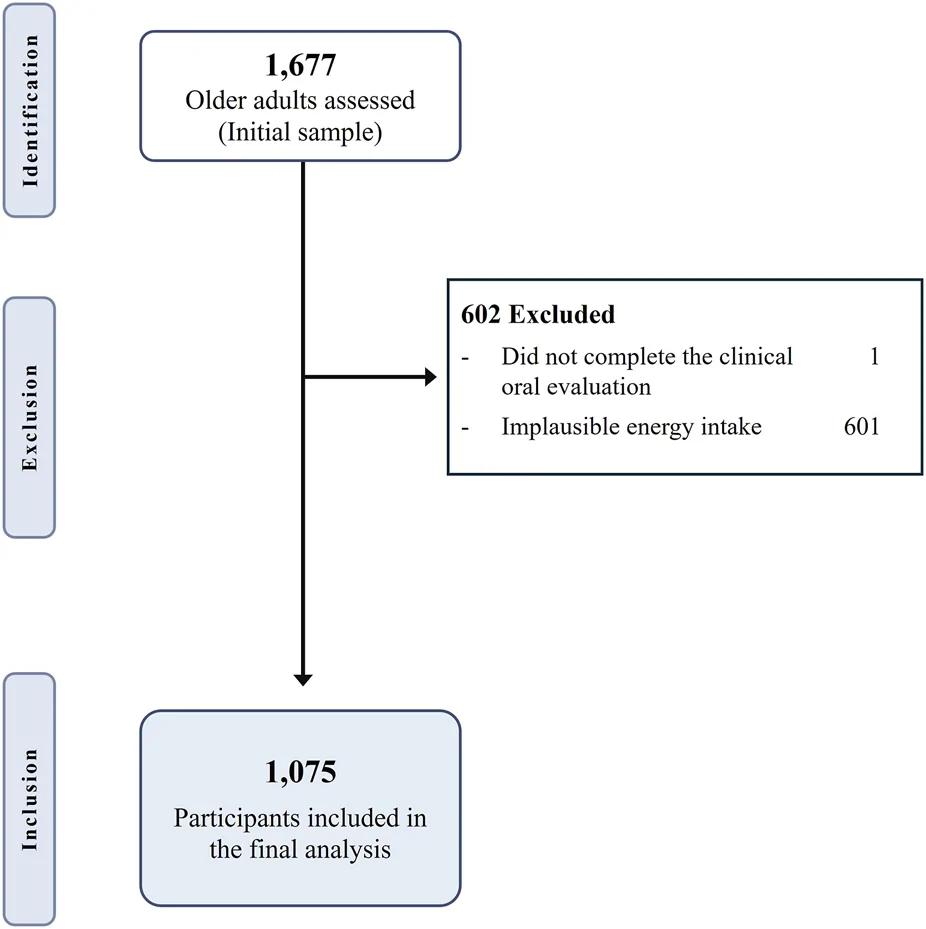
Flowchart of participants showing eligibility assessment and exclusion criteria.

### 2.2 Variables

To measure the variables of interest, all participants attended a previously scheduled appointment. During this visit, trained personnel conducted a structured interview that included the collection of sociodemographic data (age, sex, education, and marital status). Dietary intake was assessed using a semi-quantitative food frequency questionnaire (FFQ) comprising 110 items that covered food consumption over the previous year. For each item, information was collected on frequency (days per week), number of times per day, portion size, and number of portions. Daily intake (grams/day) for each food item was estimated from the reported frequency and portion size. Energy, macro-, and micronutrient intake were then estimated using the USDA FoodData Central food composition database. For each food reported in the FFQ, the corresponding entry in the database was identified based on the closest available match to the food consumed by the study population; when an exact match was unavailable, the most compositionally comparable food item was selected. The same food-composition source and matching approach were applied consistently across participants. USDA FoodData Central was selected because it provided information for a broad range of dietary components required to calculate the Dietary Inflammatory Index (DII). Body mass index (BMI) was calculated from weight and height measurements obtained with a calibrated electronic scale and a portable stadiometer, respectively, in accordance with the standardized protocols of the National Institute of Public Health in Mexico. Cognitive status was assessed using the Mini-Mental State Examination (MMSE).

### 2.3 Oral examination

Tooth loss and/or presence of teeth were evaluated. The evaluation was performed with the patient seated in a chair, using a headlamp, a No. 5 mirror, and an IPC probe (WHO type). A dental surgeon previously standardized for this purpose performed the assessment. Tooth loss (absence of one or more teeth in the mouth) was recorded. We also noted whether participants had functional dentition, defined as at least 20 teeth present in the mouth, considering the minimum number required to perform functions such as mastication (Fueki and Baba, 2017; Yan et al., 2023).

### 2.4 Dietary inflammatory index (DII) calculation

The DII was energy-adjusted and calculated from participants’ responses to a food-frequency questionnaire, following the methodology described by Shivappa et al. (2014). Briefly, DII was estimated by creating a z-score for each food parameter in the index. This involved subtracting the individual’s current intake from the global mean provided by the representative world database, then dividing the result by the standard deviation (SD). The z-score values were transformed using the empirical cumulative distribution function, then multiplied by 2 and adjusted by subtracting 1 to obtain a symmetrical distribution centered at 0. The resulting values were then multiplied by the inflammatory score assigned to each food parameter. Finally, the scores for all individual food parameters were summed to calculate the DII (Rivera-Paredez et al., 2022). Among the 45 food parameters, 38 were available in the FFQ, including total energy, carbohydrates, proteins, alcohol, fiber, total fats, cholesterol, saturated fat, fatty acids, monounsaturated polyunsaturated fatty acids, trans fat, omega 3, omega 6, caffeine, iron, magnesium, niacin, riboflavin, selenium, thiamine, and vitamins A, C, D, E, B6, and B12, as well as flavonoids. The following items were not available: rosemary, turmeric, ginger, saffron, eugenol, thyme, and green/black tea. However, these parameters are not frequently consumed by the Mexican population (Rivera-Paredez et al., 2022). To adjust DII for energy, we used the residual method to estimate total energy intake. DII groups were divided using an interquartile split (Q1 = bottom quartile 25%, Q2 = middle 50%, Q3 = top quartile 25%) for statistical analysis.

### 2.5 Ethical considerations

This study was approved by the National Committee for Scientific Research and the Ethics Committee on Health Research (CEI-009-20160601) of IMSS. The work described complies with the World Medical Association Code of Ethics (the Declaration of Helsinki) and the guidelines of Mexico’s Ley General de Salud. All participants signed a consent form.

### 2.6 Statistical analysis

Descriptive statistics and percentage distributions were calculated using IBM SPSS (Statistical Package for the Social Sciences) version 24.0 for Windows. The Pearson’s Chi Square test was used to determine the uniformity of the frequency and distribution for comparisons of group number of teeth and categorical variables were presented as frequencies and percentages (age, education level, marital status, smoking, diabetes mellitus and hypertension diagnosis) or mean and standard deviation were used for continuous variables (Dietary Inflammatory Index, Body Mass Index and Mini Mental State Examination). We assessed normality using the Kolmogorov-Smirnov test and compared groups for the number of teeth and the daily intake of fiber, carbohydrates, sugar, and cholesterol using the Kruskal–Wallis test. Finally, we conducted an ordinal logistic regression analysis to examine the association between the Dietary Inflammatory Index and the number of teeth adjusted for age, education, marital status, smoking status, and cognitive impairment. We set the Q1 group as the reference category. Covariates were selected *a priori* based on their established associations with both tooth retention and dietary intake in the literature. Statistical significance was set at p < 0.05.

## 3 Results

From the initial sample of 1,677 older adults, 1,075 participants were included in the final analysis after excluding individuals who did not complete the interview or clinical oral evaluation, or who had an implausible energy intake (<500 or > 6,500 kcal/day) (Figure 1). The sociodemographic characteristics of these participants are presented in Table 1. Significant differences were observed across several variables related to tooth number, including age, education level, marital status, smoking status, cognitive impairment, and DII (all p < 0.05). Participants with more teeth were younger (65.68 ± 4.99 years), while participants in the edentulous group had a higher mean age (70.25 ± 7.45 years). Significant differences were observed across tooth-loss groups for educational level and marital status. Participants with more natural teeth had higher educational attainment and were more frequently married or living in a free union, whereas lower educational attainment, widowhood, and divorced/separated status were more common among those with fewer teeth and among edentulous participants (p < 0.05). Smoking also showed significant differences between the groups (p = 0.002). The proportion of smokers was higher among participants with 1–10 teeth (57.5%) and among edentulous participants (63.9%) than among those with 21–28 teeth (44.9%). In contrast, no significant differences in the prevalence of diabetes mellitus and hypertension were observed between the groups (p = 0.231; p = 0.162). Regarding MMSE scores, participants in the 21-28 teeth group (25.43 ± 2.56) had significantly higher scores than those in the 1-10 teeth group (24.95 ± 2.59) and the edentulous group (24.77 ± 3.01). Compared with the group with 11–20 teeth (24.93 ± 3.05), the edentulous group showed significantly lower MMSE scores (24.77 ± 3.01).

**TABLE 1:** Characteristics of study participants, overalls and according to the number of natural teeth.

Characteristic	Overall	28–21	20/Nov	10/Jan	Edentulous	p-value
	​	Natural teeth	Natural teeth	Natural teeth	​	
	% (n)	% (n)	% (n)	% (n)	% (n)	
​	1,075 (100.0)	51.6 (559)	29.6 (317)	11.9 (127)	6.9 (72)	
Age (years, mean ± SD)	66.88 ± 5.82	65.68 ± 4.99	67.58 ± 6.18	68.51 ± 5.88	70.25 ± 7.45	**< 0.001**
Sex						
Male	52.6 (565)	53.5 (299)	51.7 (164)	48.0 (61)	56.9 (41)	0.598
Female	47.4 (510)	46.5 (260)	48.3 (153)	52.0 (66)	43.1 (31)	
Education						
No schooling	1.8 (19)	1.8 (10)	2.2 (7)	0.8 (1)	1.4 (1)	**0.002**
1–6 years	19.5 (210)	15.2 (85)	21.1 (67)	30.7 (39)	26.4 (19)	
≥7 years	78.7 (846)	83.0 (464)	76.7 (243)	68.5 (87)	72.2 (52)	
Marital status						
Single	14.0 (151)	16.1 (90)	10.7 (34)	13.4 (17)	13.9 (10)	**<0.001**
Free union or married	61.8 (664)	65.8 (368)	62.1 (197)	52.8 (67)	44.4 (32)	
Divorced-separated	11.7 (126)	8.8 (49)	13.6 (43)	15.7 (20)	19.4 (14)	
Widowed	12.5 (134)	9.3 (52)	13.6 (43)	18.1 (23)	22.2 (16)	
Smoking status						
Never (no)	52.2 (561)	55.1 (308)	54.6 (173)	42.5 (54)	36.1 (26)	**0.002**
Current (yes)	47.8 (514)	44.9 (251)	45.4 (144)	57.5 (73)	63.9 (46)	
Diabetes mellitus						
Without	77.1 (829)	79.6 (445)	75.1 (238)	73.2 (93)	73.6 (53)	0.231
With	22.9 (246)	20.4 (114)	24.9 (79)	26.8 (34)	26.4 (19)	
Hypertension						
Without	56.3 (605)	59.4 (332)	50.8 (161)	53.5 (68)	61.1 (44)	0.162
With	43.7 (470)	40.6 (227)	49.2 (156)	46.5 (59)	38.9 (28)	
MMSE (mean ± SD)	25.18 ± 2.76	25.43 ± 2.56	24.93 ± 3.05	24.95 ± 2.59	24.77 ± 3.01	**0.022**
DII (mean ± SD)	−3.46 ± 0.76	−3.55 ± 0.75	−3.48 ± 0.71	−3.21 ± 0.73	−3.17 ± 0.99	**<0.001**
BMI (kg m^−2^, mean ± SD)	28.23 ± 4.64	28.04 ± 4.32	28.42 ± 5.06	28.61 ± 4.75	28.15 ± 4.97	0.498

Mini-Mental State Examination (MMSE), Diet Inflammatory Index (DII), (BMI Body Mass Index). Values in bold are statistically significant.

### 3.1 Dietary inflammatory index associated with tooth count and functional dentition

Groups were generated based on an interquartile split of DII and compared with respect to the number of teeth present. The Chi-square test showed statistically significant differences between these groups. As dietary patterns became more pro-inflammatory, the proportion of individuals with complete functional dentition decreased. At the same time, the proportion with severe tooth loss or edentulism increased (Table 2).

**TABLE 2:** Comparison between the number of teeth and DII.

DII	Overall	21-28 teeth	11-20 teeth	1-10 teeth	Edentulism	p-value
	% (n)	% (n)	% (n)	% (n)	% (n)	
Q1	29.8 (320)	32.9 (184)	31.2 (99)	16.5 (21)	22.2 (16)	**<0.001**
Q2	61.8 (664)	61.2 (342)	60.6 (192)	67.6 (86)	61.1 (44)	
Q3	8.5 (91)	5.9 (33)	8.2 (26)	15.7 (20)	16.7 (12)	

Values in bold are statistically significant.

Within the total sample, 70.8% exhibited non-functional dentition. The analysis of functional dentition in relation to DII revealed group differences. Among those adhering to a less inflammatory diet, 67.2% had no functional dentition; for the regular diet group, 71.1% had no functional dentition, and in the more inflammatory diet group, 81.3% lacked functional dentition (Table 3).

**TABLE 3:** Functional dentition associated with DII.

DII	Absent	Present	Total	p-value
	% (n)	% (n)	n	
Q1	67.2 (215)	32.8 (105)	320	**0.032**
Q2	71.1 (472)	28.9 (192)	664	
Q3	81.3 (74)	18.7 (17)	91	

Values in bold are statistically significant.

Edentulous individuals consume significantly more carbohydrates and sugar than those with 21–28 teeth. This difference may arise from dietary adaptations to limited chewing, often involving foods like desserts, juices, or ultra-processed products. A Kruskal–Wallis test shows that tooth loss, especially edentulism, is associated with higher carbohydrate intake (Table 4). We found no statistically significant differences in fiber, sugar, or cholesterol intake between groups, regardless of the number of teeth (Table 4).

**TABLE 4:** Number of teeth present in the mouth, dietary fiber, and cholesterol consumption.

Variable	Overall	21-28 teeth	11-20 teeth	1-10 teeth	Edentulism	H	p-value
	Median	Median	Median	Median	Median		
	(25-75)	(25-75)	(25-75)	(25-75)	(25-75)		
Fiber	13.19	13.39	12.73	11.85	15.44	4.815	0.186
(g/day)	(8.67-21.29)	(8.51-21.91)	(8.69-19.85)	(8.91-20.23)	(9.30-24.77)	​	​
Cholesterol	59.99	57.19	59.2	63.02	72.69	7.360	0.061
(100 mg/day)	(36.04-113.99)	(32.43-116.18)	(36.30-107.52)	(38.43-108.81)	(49.29-145.14)	​	​
Carbohydrates	156.56	149.14	160.25	167.74	187.87	17.684	**0.001**
(150g/day)	(109.24-212.92)	(106.01-206.50)	(106.24-214.30)	(114.05-222.94)	(141.98-251.86)	​	​
Sugar	61.92	61.49	61.01	64.22	68.88	7.399	0.060
(10g/day)	(39.47-92.03)	(37.81-90.52)	(37.67-92.42)	(41.20-95.43)	(52.21-119.12)	​	​

Values in bold are statistically significant.

### 3.2 Logistic regression between the dietary inflammatory index and the number of teeth

We estimated two ordinal logistic regression models, adjusted for age, marital status, education, cognitive impairment, and smoking habits. The first model evaluated the association between the number of teeth (categorized) and the DII index categories (Table 5). We found that not having functional dentition was associated with higher odds of belonging to a more inflammatory dietary category compared with having functional dentition (OR = 1.37; 95% CI: 1.05-1.78: p = 0.020). In addition, a second ordinal logistic regression model was conducted to assess the association between the number of teeth and the dietary inflammatory index. The results indicate that having between 1 and 10 teeth (OR = 2.56: 95% CI: 1.70-3.86; p < 0.001), and being edentulous (OR = 2.13: 95% CI: 1.26-3.60; p = 0.005) were associated with higher odds of a more inflammatory diet. No significant difference was observed for the 11 to 20 teeth and 21 to 28 teeth groups, suggesting that having less teeth increases the odds of a more inflammatory diet.

**TABLE 5:** Ordinal logistic regression model.

Model 1. Number of natural teeth	Crude			Adjusted		
	OR	IC 95%	p	OR	IC 95%	p*
21-28 teeth (ref)	**1**		​	**1**		​
11-20 teeth	1.13	0.86–1.50	0.373	1.05	0.79–1.39	0.729
1-10 teeth	2.56	1.70–3.86	<0.001	2.190	1.44–3.33	<0.001
Edentulism	2.13	1.26–3.60	0.005	1.640	0.95–2.81	0.072

Model 2. Functional dentition	Crude			Adjusted		
	OR	IC 95%	p	OR	IC 95%	p*
With functional dentition (ref)	**1**		​	**1**		​
Without functional dentition	1.37	1.05–1.78	0.02	1.240	0.94–1.62	0.118

For both models, the ORs represent the odds of belonging to a more inflammatory category of the Dietary Inflammatory Index.

* The adjusted models include age, education, marital status, smoking status, and cognitive impairment.

## 4 Discussion

Oral health is increasingly recognized as a determinant of nutritional status in older adults; however, evidence linking tooth retention with the inflammatory potential of the diet remains scarce, particularly in Latin American populations. Studies indicate that adherence to unhealthy dietary patterns is associated with periodontal disease and gingival bleeding (Curca et al., 2026). This study found that as dietary patterns become more pro-inflammatory, the proportion of individuals with a complete functional dentition decreases, while the frequency of severe tooth loss or edentulism increases. This finding aligns with studies relating higher DII scores to a greater prevalence of periodontitis and tooth loss. In an analysis of NHANES data, individuals in the highest DII quartile exhibited significantly more tooth loss than those in the lowest quartile (Feng et al., 2022; Kotsakis et al., 2018; Li et al., 2021).

In the present study, subjects with more teeth and a diet with anti-inflammatory potential scored higher on the MMSE. Neurodegenerative processes have been linked to chronic low-grade inflammation; thus, diets that promote it could contribute to cognitive decline (Fang et al., 2025). Kobayashi et al. indicate that tooth loss may reduce the volume of brain regions involved in cognition, learning, and memory (Kobayashi et al., 2018). In this regard, dietary diversity and adherence to an anti-inflammatory diet could mediate the association between tooth loss and the incidence of mild cognitive impairment over 15 years (Winning et al., 2025).

Regarding sociodemographic variables, our results are consistent with current literature. Younger subjects, non-smokers, and individuals who have completed high school exhibit less tooth loss (Aguilar-Díaz et al., 2021; Wu et al., 2025; Kawahara et al., 2021). A study indicates that individuals with higher levels of education have, on average, 18% fewer missing teeth than those with no schooling (Aguilar-Díaz et al., 2021). Moreover, studies have shown an association between income inequality and oral health. Proposed explanations include limited access to preventive dental care and social resources, as well as low-income-related behaviors such as smoking, high sugar intake, infrequent dental visits, and poor oral hygiene (Ferreira et al., 2020).

Several factors can influence food choices, including economic, social, food security, and health considerations, as well as oral and masticatory function. The presence and distribution of teeth can affect the mechanical processing of foods, and tooth loss, particularly in posterior teeth, may be associated with difficulties consuming foods that require greater chewing effort, such as raw vegetables, fruits, nuts, and some meats (McGowan et al., 2020; Mikami et al., 2024; van de Rijt et al., 2021). These functional limitations may contribute to changes in food selection and dietary patterns, favoring softer, more refined, and energy-dense foods that are easier to chew but tend to have a higher inflammatory potential. This mechanism offers a plausible explanation for reverse causality in our findings: rather than diet quality determining tooth retention, impaired masticatory function from tooth loss may drive higher DII scores. Given the cross-sectional design, we cannot determine whether preserving natural dentition facilitates healthier food choices, pro-inflammatory dietary patterns contribute to tooth loss, or both. Nonetheless, this interrelation highlights oral health as a factor that warrants consideration in nutritional assessment strategies for aging populations. Our findings support the oral–nutrition relationship, suggesting that oral health should be integrated into nutritional assessment and intervention strategies for older adults. Given the recognized role of chronic low-grade inflammation in frailty, sarcopenia, and cognitive decline, our findings provide evidence of a close link between oral health and inflammatory dietary exposure in older adults, one that may be overlooked in nutritional assessment and warrants further longitudinal investigation to clarify its direction.

## 5 Conclusion

Greater tooth retention and the presence of functional dentition were associated with lower dietary inflammatory potential in older adults. These findings support an association between tooth retention and dietary inflammatory patterns, underscoring the relevance of integrating oral health into nutritional assessment and public health strategies. However, the cross-sectional design does not allow the temporal or causal direction of this association to be established. Two mechanisms are plausible and not mutually exclusive: pro-inflammatory dietary patterns may increase susceptibility to periodontal disease and tooth loss; alternatively, tooth loss and masticatory impairment may shift diets toward softer, more processed, and more inflammatory foods. Longitudinal studies are needed to disentangle this potentially bidirectional relationship between tooth loss and dietary patterns.

The absence of broader oral health and functional measures is a limitation. Therefore, it is important to assess other risk factors that could influence the possible association and that were not measured in our study, particularly periodontal status, which cannot be ruled out. Although we assessed tooth count and functional dentition, we did not evaluate periodontal status, dental caries, occlusal support, prosthetic status and use, masticatory performance, chewing ability, or oral health-related quality of life. These factors may influence food selection and intake, thereby contributing to the association between tooth retention and dietary inflammatory potential. Future studies should include comprehensive oral health and functional assessments to clarify the mechanisms linking oral health and dietary patterns.

## Data Availability

The raw data supporting the conclusions of this article will be made available by the authors upon request, without undue reservation.
