# Supplementary material for: Association between tooth retention and lower inflammatory dietary pattern in older adults
Source: Front Aging. 2026 Oct 2;7:1945744. doi: 10.3389/fragi.2026.1945744 (PMC13672328; doi:10.3389/fragi.2026.1945744)
Supplement: Supplementary file 1 [file Table1.docx]

**Supplementary Table**

**Table 1. Characteristics of the study participants, overalls and according the number of natural teeth**

| Characteristics participants | Overall | 28-21 | 20-11 | 10-1 | Edentulous | p-value |
| --- | --- | --- | --- | --- | --- | --- |
|  |  | natural teeth | natural teeth | natural teeth |  |  |
|  | % (n) | % (n) | % (n) | % (n) | % (n) |  |
|  | 1677 (100.0) | 51.6 (866) | 29.6 (496) | 11.9 (199) | 6.9 (116) |  |
| Age, years  (mean ± SD) | 66.22 ± 6.04 | 65.87 ± 4.97 | 67.86 ± 6.30 | 69.06 ± 6.37 | 71.47 ± 8.10 | < 0.001** |
| **Sex (%)** |  |  |  |  |  |  |
| Male | 49.9 (837) | 51.6 (447) | 48.2 (239) | 46.7 (93) | 50.0 (58) | 0.493* |
| Female | 50.1 (840) | 48.4 (419) | 51.8 (257) | 653.3 (106) | 50.0 (58) |  |
| **Education (%)** |  |  |  |  |  |  |
| No schooling | 1.4 (23) | 1.4 (12) | 1.8 (9) | 0.5 (1) | 0.9 (1) | <0.001* |
| 1-6 years | 19.1 (321) | 14.4 (125) | 20.0 (99) | 32.7 (65) | 27.6 (32) |  |
| ≥ 7 years | 79.5 (1333) | 84.2 (729) | 78.2 (388) | 66.8 (133) | 71.6 (83) |  |
| **Marital status (%)** |  |  |  |  |  |  |
| Single | 14.1 (237) | 15.9 (138) | 10.7 (53) | 14.6 (29) | 14.7 (17) | <0.001* |
| Free union or married | 60.8 (1020) | 65.1 (564) | 59.5 (295) | 54.8 (109) | 44.8 (52) |  |
| Divorced-separated | 11.7 (197) | 9.6 (83) | 13.3 (66) | 14.6 (29) | 16.4 (19) |  |
| Widowed | 13.3 (223) | 9.4 (81) | 16.5 (82) | 16.1 (32) | 24.1 (28) |  |
| **Smoking status (%)** |  |  |  |  |  |  |
| Never (No) | 51.9 (871) | 54.3 (470) | 53.8 (267) | 44.2 (88) | 39.7 (46) | 0.002* |
| Current (Yes) | 48.1 (806) | 45.7 (396) | 46.2 (229) | 55.8 (111) | 60.3 (70) |  |
| **Diabetes Mellitus (%)** |  |  |  |  |  |  |
| Without | 78.0 (1308) | 81.4 (705) | 75.0 (372) | 74.9 (149) | 70.7 (82) | 0.004* |
| With | 22.0 (369 | 18.6 (161) | 25.0 (124) | 25.1 (50) | 29.3 (34) |  |
| **Hypertension (%)** |  |  |  |  |  |  |
| Without | 59.9 (1005) | 62.7 (543) | 55.4 (275) | 59.8 (119) | 58.6 (68) | 0.071* |
| With | 40.1 (672) | 37.3 (323) | 44.6 (221) | 40.2 (80) | 41.4 (48) |  |
| **Cognitive impairment (MMSE)** (mean ± SD) | 25.19 ± 2.78 | 25.38 ± 2.56 | 25.23 ± 2.92 | 24.69 ± 2.79 | 24.45 ± 3.49 | <0.001** |
| **Diet Inflammatory Index (DII)** (mean ± SD) | 2.69 ± 2.09 | 2.80 ± 2.01 | 2.65 ± 2.20 | 2.50 ± 2.08 | 2.35 ± 2.20 | 0.061** |
| **Body Mass Index** (kg m^−2^, mean ± SD) | 28.12 ± 4.69 | 27.83 ± 4.27 | 28.57 ± 5.21 | 28.27 ± 4.80 | 28.11 ± 5.02 | 0.046** |
| *Chi-square, **ANOVA |  |  |  |  |  |  |
